# Trapezius Motor Evoked Potentials From Transcranial Electrical Stimulation and Transcranial Magnetic Stimulation: Reference Data, Characteristic Differences and Intradural Motor Velocities in Horses

**DOI:** 10.3389/fnins.2022.851463

**Published:** 2022-04-27

**Authors:** Sanne Lotte Journée, Henricus Louis Journée, Hanneke Irene Berends, Steven Michael Reed, Wilhelmina Bergmann, Cornelis Marinus de Bruijn, Cathérine John Ghislaine Delesalle

**Affiliations:** ^1^Equine Diagnostics, Wyns, Netherlands; ^2^Research Group of Comparative Physiology, Department of Translational Physiology, Infectiology and Public Health, Faculty of Veterinary Medicine, Ghent University, Merelbeke, Belgium; ^3^Department of Neurosurgery, University of Groningen, University Medical Center Groningen, Groningen, Netherlands; ^4^Department of Orthopedic Surgery, University Medical Center Utrecht, Utrecht University, Utrecht, Netherlands; ^5^Department of Orthopedics, Amsterdam University Medical Center, Amsterdam, Netherlands; ^6^Rood and Riddle Equine Hospital, Lexington, KY, United States; ^7^Department of Veterinary Science, Maxwell H. Gluck Equine Research Center, University of Kentucky, Lexington, KY, United States; ^8^Division of Pathology, Department of Biomolecular Health Sciences, Faculty of Veterinary Medicine, Utrecht University, Utrecht, Netherlands; ^9^Wolvega Equine Clinic, Oldeholtpade, Netherlands

**Keywords:** transcranial stimulation, trapezius, multifidus, spinal accessory nerve, horses, central conduction velocity, motor evoked potentials

## Abstract

**Reason for Performing Study:**

So far, only transcranial motor evoked potentials (MEP) of the extensor carpi radialis and tibialis cranialis have been documented for diagnostic evaluation in horses. These allow for differentiating whether lesions are located in either the thoraco-lumbar region or in the cervical myelum and/or brain. Transcranial trapezius MEPs further enable to distinguish between spinal and supraspinal located lesions. No normative data are available. It is unclear whether transcranial electrical stimulation (TES) and transcranial magnetic stimulation (TMS) are interchangeable modalities.

**Objectives:**

To provide normative data for trapezius MEP parameters in horses for TES and TMS and to discern direct and indirect conduction routes by neurophysiological models that use anatomical geometric characteristics to relate latency times with peripheral (PCV) and central conduction velocities (CCV).

**Methods:**

Transcranial electrical stimulation-induced trapezius MEPs were obtained from twelve horses. TES and TMS-MEPs (subgroup 5 horses) were compared intra-individually. Trapezius MEPs were measured bilaterally twice at 5 intensity steps. Motoneurons were localized using nerve conduction models of the cervical and spinal accessory nerves (SAN). Predicted CCVs were verified by multifidus MEP data from two horses referred for neurophysiological assessment.

**Results:**

Mean MEP latencies revealed for TES: 13.5 (11.1–16.0)ms and TMS: 19.7 (12–29.5)ms, comprising ∼100% direct routes and for TMS mixed direct/indirect routes of L:23/50; R:14/50. Left/right latency decreases over 10 > 50 V for TES were: –1.4/–1.8 ms and over 10 > 50% for TMS: –1.7/–3.5 ms. Direct route TMS-TES latency differences were 1.88–4.30 ms. 95% MEP amplitudes ranges for TES were: L:0.26–22 mV; R:0.5–15 mV and TMS: L:0.9 – 9.1 mV; R:1.1–7.9 mV.

**Conclusion:**

This is the first study to report normative data characterizing TES and TMS induced- trapezius MEPs in horses. The complex trapezius innervation leaves TES as the only reliable stimulation modality. Differences in latency times along the SAN route permit for estimation of the location of active motoneurons, which is of importance for clinical diagnostic purpose. SAN route lengths and latency times are governed by anatomical locations of motoneurons across C2-C5 segments. TES intensity-dependent reductions of trapezius MEP latencies are similar to limb muscles while MEP amplitudes between sides and between TES and TMS are not different. CCVs may reach 180 m/s.

## Introduction

Since 1996, the recording of motor evoked potentials (MEP) that are elicited by transcranial stimulation has been incorporated in the diagnostic work-up of horses suspected of suffering from spinal cord injury (Mayhew and Washbourne, 1996; Nollet et al., 2003b,^2004^). In addition to neurological examination, the technique is complementary to medical anatomical imaging techniques, and additional diagnostic tests such as blood work and cerebrospinal fluid analysis (Nollet et al., 2003a,^2004^, ^2005^; Journée et al., 2019; Rijckaert et al., 2019). For decades, it has been proven in human medicine that transcranial MEPs are sensitive to even minor damage to the spinal cord. Therefore, their signal output is rated for monitoring spinal functional integrity during corrective surgery and removal of spinal cord tumors. Likewise, it has been well described that transcranial MEP curve parameters have a highly predictive value for post-operative neuronal functional ability of human patients (MacDonald et al., 2013). In horses, transcranial electric (TES) and magnetic (TMS) stimulation are well known as non-invasive diagnostic tests with low discomfort, under sedated conditions (Nollet et al., 2003a; Journée et al., 2015, 2018). Both techniques are able to discern between presence or absence of possible neurological lesions. They also allow to discriminate between either a spinal or supraspinal lesion location and to identify an either focal or widespread presence of lesions. A pathological condition that triggers widespread damage of neural tissues can for example be seen with many infectious diseases and dietary insufficiencies, such as equine degenerative myeloencephalopathy (EDM) (Nollet et al., 2002, 2003c,^2005^; Rijckaert et al., 2019).

**FIGURE 1:**
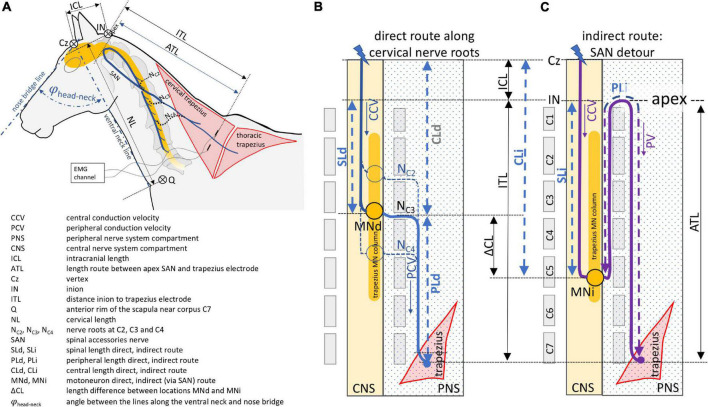
Schematic overview of the direct **(B)** and indirect **(C)** neuroanatomic pathways between transcranial stimulation and recording electrodes inserted into the trapezius muscle. The direct route courses along three cervical nerves and the indirect route via the spinal accessory nerve. The neuroanatomy and definitions of geometric measures and symbols are illustrated and explained in panel **(A)**.

So far, only TES and TMS induced muscle MEPs of the musculus (m.) extensor carpi radialis (ECR) and m. tibialis cranialis (TC) have been documented for diagnostic evaluation in horses (Mayhew and Washbourne, 1996; Nollet et al., 2003a,^2004^, ^2005^; Rijckaert et al., 2019). These MEPs allow for differentiating whether lesions are located in either the thoraco-lumbar region or the cervical myelum and/or the brain. A further differentiation between the cervical spine and brain is possible when evaluating MEP recordings from high cervical innervated muscle groups. An attractive choice for this purpose, is the trapezius muscle. Trapezius MEPs are conducted *via* C2-C4 cervical nerve roots and are recorded with a similar convenience as MEPs from the TC and ECR muscles by means of surface or subcutaneous needle electrodes (Journée et al., 2020a,b). The electrodes can be placed at a sufficient distance from the TMS coil to avoid interference by magnetic pulses of varying strength leading to possible saturation and blockage of connected amplifiers. The trapezius muscle has a complex nerve supply around the plexus cervicalis, including both a short direct and a long indirect conduction route as depicted in the diagram in Figure 1A.

The short conduction route runs along cervical spinal nerves C2, C3, and C4 toward the trapezius muscle (Gavid et al., 2016). The indirect conduction route follows a detour *via* the spinal accessory nerve (SAN) which originates from motoneurons located in the ventrolateral column of the lateral part of the ventral gray horn located between C2 and C5 in human and most animals, including horses. In rats, however, it extends to the rostral quarter of C6 (Flieger, 1966; Routal and Pal, 2000; Ullah et al., 2007; Boehm and Kondrashov, 2016). In this indirect conduction route, the motor axons ascend all the way up through the foramen magnum reaching the apex and emerge together with cranial nerves IX and X through the jugular foramen from where they enter the cervical plexus to join the axons of C2, C3, and sometimes C4 roots from the short direct route on its course to the trapezius muscle (Tubbs et al., 2011; Gavid et al., 2016). When both pathways are functional, the conduction times along the direct route of the cervical nerves will be the shortest and govern the MEP latency times regardless if the SAN is the most prominent supplier. The contribution of each cervical nerve varies between patients as has been shown intraoperatively in humans by selective supramaximal stimulation. According to Kim et al. (2014), the trapezius muscle receives a motor input from C4 in 83%, from C3 in 46% and from C2 in 8% of cases. Lower or comparable percentages are found from cervical root stimulation while the SAN route yields as main supplier the highest response percentages (Pu et al., 2008; Svenberg Lind et al., 2015; Gavid et al., 2016; Brînzeu and Sindou, 2017). Up until now, these data are unknown in horses. Although TES and TMS are interchangeable techniques for assessment of ECR and TC muscle MEPs, MEP latencies do show differences of at least one synaptic delay (Amassian et al., 1989; Edgley et al., 1990; Burke et al., 1993; Ubags et al., 1999; Journée et al., 2020a). However, it is not known whether both techniques are interchangeable for assessment of trapezius muscle MEPs.

When latencies from direct and indirect routes can be discerned and magnitudes of route lengths inside and outside the dura are assessed and peripheral nerve conduction velocities are known, this theoretically allows to locate functional active motoneurons in the spinal cord and to estimate central motor velocities. In that way, differences in latency times along the SAN route permit to estimate the location of active motoneurons, which is of importance from a clinical diagnostic point of view.

The objectives of the current study were to provide normative data for trapezius MEP parameters in horses for TES and TMS, to discern direct and indirect conduction routes by application of neurophysiological models that use anatomical geometric characteristics to relate latency times with peripheral conduction velocities (PCV) and to determine intradural central conduction velocities (CCV).

## Materials and Methods

The TES group consisted of twelve healthy warmblood horses including 6 geldings and 6 mares, aged: 10.7 ± 5.5 years (mean ± SD). No abnormalities were found during clinical neurological examination. The height at withers was 160.8 ± 10 centimeters (mean ± SD). The subgroup of the TMS study consisted of 5 horses (also part of the TES group) (3 geldings, 2 mares) aged: 11.1 ± 7.1 years with a height at withers of 160.0 ± 7.0 cm.

The study protocol was approved by the animal ethics committee of the University of Groningen, the Netherlands and registered as DEC6440A and DEC6440B for the study of reference data of TES and TMS. Results of the TES and TMS studies for the TC and ECR MEPs were previously published (Journée et al., 2018, 2020a).

To ascertain the central conduction velocities predicted by the model in the current study in practice, CCV’s were derived from TES-MEP latency differences of multifidus muscles at intercorporal levels of C2-3 and C5-6 in two mares. Both horses were referred for neurophysiological evaluation and deemed healthy. Being monosegmentally innervated by short peripheral nerve roots of about equal lengths, the multifidus muscle was considered as an optimal choice for measuring the central conduction velocity. The muscle potentials were recorded by long needle electrodes in warmblood mares with ages of 2 and 4 years and heights of 1.53 and 1.67 m, respectively. The calculated CCV across the enclosed segments resulted from division of the bridged intersegmental length by the latency difference.

### Methods of Measurement

Horses were prepared as previously described (Journée et al., 2015). Sedation was performed in all horses, each time by intravenous administration of detomidin (Detosedan)^1^ and butorphanol (Butomidor)^2^ (both 1.5–2.0 μg/kg in total).

A subcutaneous ringblock surrounding the vertex Cz of about ∅ 8 cm was placed, using 300–400 mg lidocaine 2% + adrenaline.

For TES, 2 needle electrodes (length 35 mm, diameter 0.45 mm, type RMN35/0.45 Electrocap BV, Nieuwkoop, The Netherlands) were placed subcutaneously in frontal direction 2.5 cm bilateral from the vertex at Cz. TES was performed using biphasic multipulse trains of 3 pulses (constant voltage, interpulse interval ipi = 1.3 ms), using a human intraoperative neurophysiological monitoring system (Neuro-Guard JS Center, Bedum, Netherlands).

Application of a voltage series consisting of 10 V steps starting at 0V was selected out of the original voltage scheme applied in a previous study (Journée et al., 2018). TES was performed twice at each voltage. After reaching the transcranial electrical motor thresholds (ET) the stimulation was continued to ET + 50V. The transcranial stimulation threshold was defined at stimulation intensity (V for TES and % of maximum output for TMS) at the first occurrence of the early muscle MEP after the latency jump from the late to the early MEP (Journée et al., 2020a).

Transcranial magnetic stimulation was applied through a circular coil (MC 125, Medtronic Functional Diagnosis A/S, Skovlunde, Denmark, maximum magnetic gradient 4 1kT/s) placed symmetrically over the midline of the head with the lower rim about 2 cm frontal from the vertex (Cz) being connected to a MagPro Compact magnetic stimulator (Medtronic Functional Diagnosis A/S). Biphasic pulses of 0.28 ms length were applied using a 10% stepwise increasing protocol, starting at 0%. TMS was performed twice at each step. When TMS motor thresholds (MT) were reached, which was detected at the stepwise latency reduction at the transition from extracranial to intracranial MEPs (Journée et al., 2020a), stimulation was continued to MT + 50%.

Motor evoked potentials were recorded simultaneously and bilaterally from the extensor carpi radialis (ECR), tibialis cranialis (TC) and trapezius muscles. The MEP data of the ECR and TC are described in a previously published study (Journée et al., 2020a) and are used for comparison in the current study. The trapezius MEPs were recorded from subcutaneous needle electrodes (82015-PT L 12 mm 27GA Rochester Lutz, FL, United States) inserted into the cervical part of the trapezius muscle, about 20 cm cranial from the middle of the rim of the scapula (see Figure 1A). A ground needle electrode was placed subcutaneously in the neck. The distance between the electrodes was about 20 cm. The signals were band pass filtered between 50 and 2500 Hz (3dB cut-off level) and digitally stored for later processing. The sample frequency was 4.3 kHz/channel. The used sample frequency for the multifidus MEPs for the CCV measurements was 8.6 kHz/channel which agreed with a time resolution between samples of 0.12 ms. Axonal lengths were estimated by determining distances, using a tape measure, between specific anatomic landmarks for different conduction trajectory sections: The intracranial axonal length (ICL) is assigned as the distance between Cz and the inion (IN), the cervical length (NL) is assigned as the distance between the inion and the anterior rim of the scapula (Q) near the corpus of C7. The measures were taken with the head in a relaxed neutral position enclosing a head-neck angle φ_head–neck_ of approximately 80 degrees between the lines along the ventral surface of the neck and the bridge of the nose as depicted in Figure 1A.

Additional anatomical distances are described in the legend of Figure 1 and the trapezius electrode closest to the inion. The peripheral motor axons either follow straight routes from the cervical nerves C2, C3 and C4 to the cranial electrode or they follow a detour route *via* the SAN. As mentioned previously, this indirect route starts at the rootlets of the myelum coursing between C2 and C5, passes the foramen magnum and returns after reaching the apex *via* the foramen jugularis and finally heads to the trapezius muscle. The straight direct route is depicted in a schematic overview in Figure 1B. PLd is the peripheral axonal length of the direct route. The indirect route is depicted schematically in Figure 1C. PLi is the length of the indirect route. The schematic drawings show a motoneuron trajectory in the myelum (yellow column in Figures 1B,C) starting slightly above C2 level and extending to the rostral part of C5. Important to notice is that the direct and indirect routes do not necessarily share the same active motoneurons. These conduction routes are indicated by MNd and MNi and their difference in length is indicated by △CL. CL is the length of the spinal motor tracts between IN and the cervical segment exit location of that specific conduction route. CL is indexed as CLd or CLi for the direct or indirect route, respectively.

### Data Processing

#### Motor Evoked Potential Parameters

For both TES and TMS, considered MEP parameters were motor latencies (EL and ML), conduction velocities and amplitudes of the recorded trapezius muscle MEPs. Wave morphology of MEPs was not considered in the current study. The parameters were subjected to left vs. right comparisons and to reveal the dependence of motor latencies and MEP amplitudes on stimulation intensities by regression analysis.

The motor latencies are defined as the time lag between the onsets of the stimulation artifact of the TMS pulse or TES pulse train and generated MEPs when these were unambiguously distinguishable from baseline noise. The electrical conduction velocities (ECV) for TES, and magnetic conduction velocities (MCV) for TMS are compound velocities that include both central and peripheral axonal conduction velocities. Net axonal conduction times and velocities were estimated by correction for interneuron and neuromuscular synaptic delays of 1.5 ms and 1 ms, respectively, as previously described (Journée et al., 2020a). In addition, the dependence of conduction times and compound conduction velocities were determined as function of the time lag t_epsp_ between the TES induced onset of the build-up of excitatory potentials (epsp) and neuron firing.

The MEP amplitude was defined as the maximum amplitude differences (top-top values) within the transcranial time window as defined by the time region before the onset of the subthreshold late MEP, just before the stepwise transition to the transcranial MEP.

#### Identification of Involved Conduction Pathways From Direct and Indirect Latency Times

The models depicted in Figures 1B,C and their associated conduction route lengths were used to relate recorded MEP latency times to either the indirect or direct conduction route, represented by Ti and Td. In case the direct and indirect conduction route share the same motoneurons the segmental distance between active motoneurons (△CL) as defined in Figure 1A is 0. Then CLd = CLi = PLi – PLd ≅ 2d_apex–MN_ where d_apex–MN_ is the distance between the apex and the shared active motoneuron MN. Subsequently, d_apex–MN_ can be computed from the difference between the indirect and direct latency times Ti and Td and the peripheral nerve conduction velocity PCV according to the equation:


(1) $$d_{\mathrm{apex}-\mathrm{MN}}=\mathrm{PCV}.\left(\mathrm{Ti}-\mathrm{Td}\right)/2$$

When Ti or Td result from different stimulation modalities (TES vs. MEP), the differences ML-EL between the TMS and TES-MEP latency times should be taken into account.

The minimum time gap between the indirect and direct route latencies Ti-Td agrees with the highest cervical location of trapezius motoneurons. When assuming d_apex–MN,min_ ≅ 15–20 cm and PCV = 90 m/s, the minimum time gap (Td-Ti)min ≅ 3.3–4.4 ms. For either transcranial stimulation modality, a mixed involvement of both direct and indirect conduction routes is expected to occur during repeated measurements. The latency distribution functions predict a silent region of at least ∼4 ms that separates direct from indirect latencies. The selection borders for MEP latencies will be established from the measured latency distribution functions.

#### Computation of Conduction Velocities

The conduction velocities are compound values of intra- and extradural (peripheral) conduction routes between vertex and trapezius muscles over an estimated total route length of ICL+ITL as depicted in Figure 1A. The intradural fraction of the conduction route was estimated from the measured dimensions of ICL, ITL and CL while equal lengths of cervical corpora were assumed. Total axonal conduction velocities were also computed when excluding synaptic delays of 2.5 ms (1 ms neuromuscular junction, 1.5 ms motoneuron) or 4.0 ms [extra 1.5 ms for the proprioceptive neuron (PN)].

### Statistical Analysis

Statistical analysis was performed with SPSS™ software, version 20.0.0, IBM™. The sequence of TES and TMS measurements series was alternated between subsequent cases to minimize time dependent bias effects on comparisons. Means were compared by paired t-tests while a significance level was set at p ≤ 5% throughout the study.

#### Motor Evoked Potential Latency Histograms

Histograms of the latency times of MEP series were composed to visualize the occurrence of direct and indirect delivered MEPs at TES intensities between ET+10 V to ET+50 V and TMS intensities between MT+10% to MT+50% for the TES-TMS subgroup (*n* = 5). These are depicted case wise (10 values per side for both TES and TMS) and combined for 5 cases (50 values/side/modality). Histograms were also derived for the total (full) TES group of 12 cases (120 values/side).

For each case n and side s, mean electrical EL_*s*,n_ and magnetic ML_*s*,n,_ with standard deviations were computed over 10 data pairs of the cervical trapezius muscles from stimulation intensities at 10–50 V above ET and 10–50% above MT.

The mean electrical and magnetic latency times per muscle group and case EL_*s*_ and ML_*s*_, with standard deviations were computed over the 12 cases for TES and 5 cases for TMS.

Mean electric and magnetic paired differences: mDL_*s*_ were computed from 50 recordings (cases, 10 values/case).

#### Dependence of Motor Evoked Potential Latencies and Amplitudes on Intensities of Transcranial Electrical Stimulation and Transcranial Magnetic Stimulation

For both sides, overall 12 TES cases and 5 TMS cases, the course of the mean latencies and amplitudes with ± 2SD confidence intervals were plotted as a function of the supra-threshold stimulation intensities from ET+10 V to +50 V for TES and MT+10% to +50% for TMS. The amplitudes were statistically processed and graphically plotted in the logarithmic domain. After back transformation to the linear domain, mean amplitudes appear as geometric means. The number of values per intensity step for the TES group (12 cases; 2 times 2 values/step and side) is 48 and the TMS group (5 cases) is 20.

For numerical evaluation, the stimulation intensity dependence of EL_*s*_‘s and ML_*s*_‘s was estimated by linear regression analysis between ET + 10 and 50 V and between MT + 10 and 50%. The computations were performed over 50 points (5 cases; 10 points/case). For the computation of the slope of the regression line and correlation, the mean MT_*s*,n_ of the stimulation intensities were subtracted from MT_*s*,n,*i*_ (i represents the stimulation intensity variable) prior to the computations.

#### Comparison of Motor Evoked Potential Latencies and Amplitudes for Transcranial Electrical Stimulation vs. Transcranial Magnetic Stimulation and Between Sides

Left-right differences for latencies and amplitudes and between the two transcranial stimulation modalities were tested on mean values by a paired *t*-test in linear and logarithmic domains, respectively.

Comparisons of mean MEP amplitudes were performed by paired *t*-tests after conversion of the amplitudes to the logarithmic domain where to our experience normal distributions are best approached (Journée et al., 2017, 2020b). The mean amplitudes were computed over the 5 individual means per case, which were obtained between supra-threshold electrical and magnetic stimulation intensities of 10 V to 50 V and 10% to MT + 50%, respectively.

#### Computation of the Accuracy of Motor Evoked Potential Latencies

The accuracy of the latency times (ACL_e_, index e refers to electrical) of TES MEPs is defined as the root of the mean squares (RMS) of the differences of latency pairs and division by their means for intensities between 10 and 50 V (50 values of 25 data pairs of 5 cases with 5 data pairs/case).

ACL_e_ is insensitive to influences of stimulation intensities on latencies and differences between cases. The normalization by division of the mean latencies makes the reproducibility ACL with exception of dependence on intensity, comparable with coefficients of variation (CV). For the accuracy for TMS, a similar computation of the accuracy ACL_m_, for magnetic latencies was used.

## Results

The stimulation protocols were successfully performed in all horses in all trapezius muscle pairs.

### Motor Evoked Potentials Resulting From Direct and Indirect Routes

**FIGURE 2:**
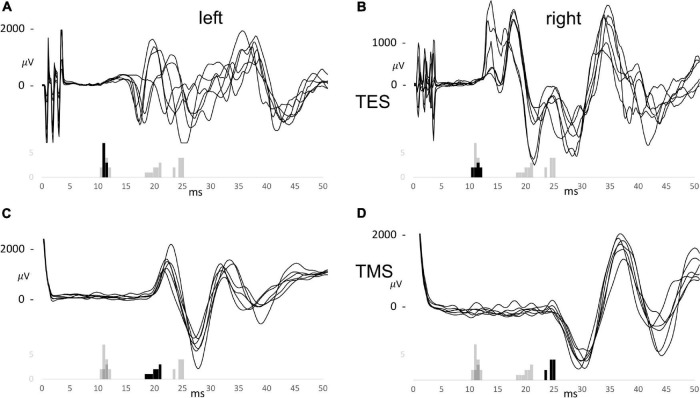
Trapezius motor evoked potential (MEP) series for TES **(A,B)** and TMS **(C,D)** for the left **(A,C)** and the right **(B,D)** sides of the first combined TES/TMS case. The histograms are overlaid and summarize the EL and ML latency times for TES between 10 and 50 V and for TMS between 10 and 50%. The black bars belong to the specific group of which 6 curves are shown. The MEP curves share the time scale of the histograms.

Figure 2 is a case example and shows six of the recorded muscle MEP series of for TES and TMS, and for both sides. These are shown with the overlaid MEP latency histograms of which the black bars refer to the latency times of the particular series of the first combined TES and TMS case. Figures 2A,B show TES MEP latency times with values ranging from 10.5 to 11 ms for the left and 10 to 11.5 ms for the right side. These only can result from conduction along the fast direct route. Likewise, the TMS-MEP latencies in Figures 2C,D are markedly longer and range from 18.5 to 21 ms for the left and 24 to 25.5 ms for the right sides. Most likely, these represent conduction along the slow indirect route. These values are markedly longer than the longest TES latency of 11.5 ms in this presented case. The mean difference between left and right for the TMS-MEP latencies of 4.69 ms implies, according to equation 1, that the active motoneurons are located on different cervical heights. The height difference is 18.7 cm. When including a bias of 4 ms of the TES to TMS difference in the same horse (Journée et al., 2020a) the range of latency values for TMS for the direct route would be well below 17 ms.

The longest TES latency of 11.5 ms would agree with 15.5 ms for TMS with the 4 ms TES to TMS latency bias. This is still 3 ms faster than the lowest recorded latency of TMS of 18.5 ms. The figure illustrates a good reproducibility of TES trapezius latencies vs. a lower reproducibility of latencies from TMS. The large TMS latency differences between sides is ascribed to large differences in indirect nerve route lengths. The small and narrow distributed latencies recorded for TES apparently indicate that the motor conduction pathways follows the direct route, traversing C2-C4 cervical roots.

### Deduction of Direct and Indirect Routes From Latency Histograms for Transcranial Electrical Stimulation and Transcranial Magnetic Stimulation

**FIGURE 3:**
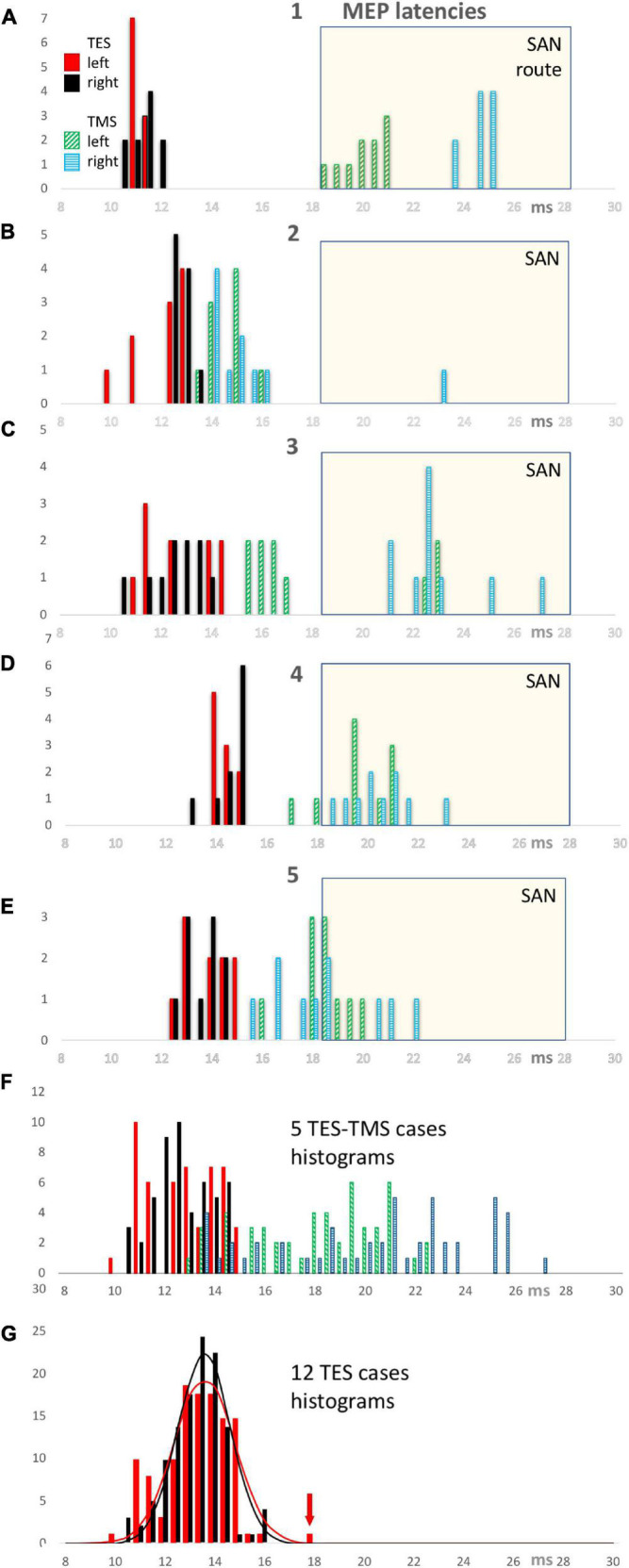
TES and TMS MEP latency time histograms of individual cases 1 through 5 (panels **A–E**) with a summarization of 5 cases in panel **(F)**. All TES latency times show a narrow distribution between 10 and 15 ms of which all observations belong to the direct motor conduction route. In contrast, the TMS latency histograms show a wide distribution between 13 and 27.1 ms representing a mixed involvement of direct and conduction indirect routes. A common SAN time window with a selection criterion of 18.5 ms is used to identify the observations which most likely represent the indirect SAN route. This window is used for the computation of the fractional involvement of direct conduction routes in the observations per case for EL and ML latencies in Table 1. All TMS latency bars of case 1 refer to indirect routes. The TMS latency bars of cases 2–5 illustrate latencies of mixed origin, both in and outside the SAN selection window. The bottom panel **(G)** shows TES MEP latency histograms of 12 cases with normal distribution functions for both sides with left in red and right in black. The red arrow points at an outlier, which, as exception, not represents the direct conduction route along the cervical nerve as do the other 119 observations.

Figures 3A–E shows the latency histograms for TES and TMS for both sides of 5 cases. Figure 3A represents the case that was also illustrated in Figure 2. Figure 3F is the summarization of all 5 cases and shows that the latency histograms for TES are zero for latencies above 15 ms. Such latencies are too short for the indirect route and can hence for 100% be ascribed to the direct route.

**TABLE 1:** Overview of fractions of the number of left and right latency times EL and ER for TES and ML and MR for TMS allocated to the direct route related to the total number of stimulations (10 per case) when using < 18.5 ms as selection criterion for TMS latencies according to the SAN time windows in Figure 3.

Direct route	EL		ER		ML		MR	
	Fraction	%	Fraction	%	Fraction	%	Fraction	%
case 1 TES/TMS	10/10	100	10/10	100	0/10	0	0/10	0
case 2 TES/TMS	10/10	100	10/10	100	10/10	100	9/10	90
case 3 TES/TMS	10/10	100	10/10	100	7/10	70	0/10	0
case 4 TES/TMS	10/10	100	10/10	100	2/10	20	0/10	0
case 5 TES/TMS	10/10	100	10/10	100	4/10	40	5/10	50
cases 1-5	10/10	100	10/10	100	23/50	46	14/50	28
cases 1-12 TES	119/120	99	120/120	100				

*The selection border for TES-MEP latencies is <16 ms.*

The SAN time window with a discrimination border at 18.5 ms was used to assign the obtained TMS induced latency times to the indirect route. This implies a TES-TMS bias of 3.5 ms. Cases 2–5 indicate situations where in 10 TMS stimulations direct and indirect routes are involved This large variability of trapezius latencies of TMS implies a poor reproducibility. This is in great contrast with the reliable reproducibility of TES latencies. The involvement of indirect routes differs markedly between cases. In case 1 only indirect SAN routes are involved in TMS while in case 2 only 1 out of 20 recorded MEPs is from the indirect route. The other 3 cases show a more mixed representation of both routes. Figure 3G shows the latency histogram for all 12 TES cases. An outlier of 18.35 ms latency time fell outside a range of 14.04–15.20 ms of the distribution of the other 19 values of that case. Since this is 3.7 ms higher than the median and more than 3 times larger than the 1.06 ms width of the distribution function, most likely this outlier represents the indirect SAN route. The four values at 16 ms in the histogram belong to 1 case and concern values between 16.12 and 16.28 ms. This is within 1 ms from the range of 13.72–15.62 ms of the remaining 16 values. Since this is too short for motor conduction of the indirect route along the SAN apex, these four values are considered to belong to the direct route. These 4 values also fall within the 95% range of the normal distribution functions illustrated in Figure 3G.

In Table 1 the fraction of MEPs allocated to the direct route (retrieved from Figures 3A–G) are listed case-wise for the TES-TMS group, together with a summary for the 5 cases and also for all 12 TES cases.

The table reveals marked differences between TES and TMS with respect to fractional representation of direct vs. indirect routes. The fractional representation of the direct route is for TES about 100% for both sides. In contrast, for TMS the direct route fractions vary per case between 0 and 100%, while the direct route fractions of the whole group of 5 cases are 46% and 28% for the left and right sides, respectively.

### Transcranial Time Window Borders

The presence of extracranial elicited MEPs below transcranial threshold intensities in the ECR and TC muscles (Journée et al., 2020a) are also encountered in trapezius MEPs. These can be masked out by a transcranial time window.

The 5 combined TES/TMS cases showed MEPs before the transcranial latency jump onsets of extracranial MEP reflex activity for TES (Journée et al., 2020a) in ranges of 30.0–38.9 ms for the left and 28.72–35.43 ms for the right side. These findings exclude presence of overlap of transcranial latency times with extracranial MEPs of maximal 27.23 ms with the lowest reflex times for TMS of 30.4 ms.

### Determination of Origin of Factors Responsible for Differences Between Motor Evoked Potential Latencies for Transcranial Electrical Stimulation vs. Transcranial Magnetic Stimulation

The origin of factors responsible for differences in latency times between TES and TMS can be classified as either peripheral or central.

(A) Factors of peripheral origin:

Motor conduction can take place along the direct and/or the indirect route, each having different conduction times.

**FIGURE 4:**
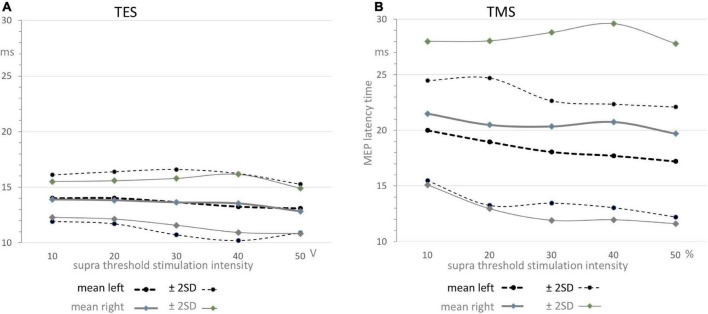
Functions of means ± 2SD confidence intervals of MEP latency times as function of supra threshold transcranial stimulation for TES **(A)** and TMS **(B)**. The graphs depicted in panel A reflect the relative narrow region between 11 and 16 ms which can solely be allocated to the direct conduction route as also illustrated by the narrow distribution functions for TES in Figure 3, while the graphs depicted in panel B illustrate the widely scattered results from TMS leading to 5–7 ms higher mean values and over 3 times widened ± 2SD confidence interval. Note that the graphs in panel A show nearby equal means while in contrast, the graphs in panel B show significant different means for TMS between left and right sides with wide confidence intervals that vary between 12 and 29 ms. The values are summarized in Table 2. All means show a gradual decrease by about –1.5 ms from 10 to 50 V for TES in panel A and about –2.5 ms for the left and –1.8 ms for the right side for TMS in panel B.

The large differences between the direct route fractions of 100% for TES and 28–46% for TMS in Table 1 are associated with significant differences in latency times. These are clearly visible in the EL and ML graphs of Figures 4A,B and summarized in Table 2. The low direct route fractions for TMS indicate mixed involvement of both conduction routes.

**TABLE 2:** Upper part: Overview of mean and standard deviations (SD) of motor latency values for TMS (ML) and TES (EL), mean ML-EL differences with significance (sig) for left (L), right (R), combined sides (L&R) and left vs. right comparisons (L-R) of trapezius muscle MEPs with mean, SD and significance.

5 combi TMS/TES cases	ML			ML-EL			EL		
	Mean ms	SD ms	sig	Mean ms	SD ms	sig	Mean ms	SD ms	sig
L	18.67	2.52		5.48	2.56	**<0.001**	13.03	0.94	
R	20.78	3.82		7.63	4.20	**<0.001**	12.96	1.28	
L&R	19.72	3.17		6.56	3.38	**<0.001**	13.00	1.16	
L-R	–2.11	2.92	**<0.001**				0.044	0.78	0.727
12 TES cases									95% range ms
L							13.56	1.23	11.10–16.00
R							13,49	1.07	11.38–15.63
L&R							13.52	1.16	11.10–16.00 widest range (is from L)

*All ML-EL differences and the ML differences between sides (L-R) are significant for p < 0.05 (marked in bold). Computations are performed over 5 combined TES/TMS cases. Lower part: normative data for TES-MEP latency times: mean, SD and 95% range of motor latency values for 12 TES cases for left, right and combined sides (L&R).*

The mixed involvement of both the direct and indirect conduction route in TMS as opposed to TES becomes evident as:

- TMS shows markedly larger mean MEP latency times being 5.48–7.63 ms longer when compared to TES. This is much longer than the around 4 ms ML-EL differences reported in ECR muscle groups of the same group of horses in a previous study (Journée et al., 2020a). These differences are also visible in Figures 4A,B.
- There is no left vs. right difference of EL’s for TES, while, in contrast, there is a highly significant difference of 2.11 ms between mean ML’s of left vs. right for TMS (Table 2).
- There are marked differences in widths of distribution of latency histograms between TMS vs. TES, with for TES a relative narrow margin of 10–16 ms compatible with involvement of only the direct route whereas the wide-spread distribution of magnetic latency times in Figure 3F is accompanied with over 3 times wider margins between 12 and 29 ms. These comprise the combination of involvement of conduction along both the direct and indirect SAN routes.
- Comparison is made with previously published ECR and TC muscle MEP parameters for both TMS and TES in the same group of horses (Journée et al., 2020a). Trapezius MEPs show clearly less pronounced differences between the standard deviations of the mean latency times for TES when compared to TMS: according to Table 2 these range for TES between 0.94 and 1.28 ms and are for TMS between 2.52 and 3.82 ms. Likewise there are more pronounced differences between CV’s of TMS induced trapezius MEPs being 7.2% (left side) or 9.9% (right) for TES. About double values of 13.5% and 18% are encountered for TMS. Similar greater differences for TMS induced ACL were found. The accuracies for TES were ACL_e_ = 2.7% (left) and 2.5% (right) and for TMS: ACL_m_ = 4.2% (left) and 5.8% (right). The differences in mean latencies, CV’s and ACL’s between TES and TMS were less pronounced for the ECR and TC muscle MEPs.

(B) Factors of central origin:

Determination of factors responsible for differences between TES and TMS MEP latency times from central origin is solely based on latency times from the direct conduction route. The trapezius ML values represent a mix of direct and indirect routes. To determine ML-EL differences from central origin, the latency times of only the direct route were used to exclude the bias from SAN detour route delays. The applied selection procedure uses *case specific discrimination levels* of mean EL_*case*_ plus a marge of 4.5 ms to avoid false inclusion of indirect conduction routes. This is more specific than the general 18.5 ms selection criterion as used in Figure 3 and Table 1. This approach precludes the possibility that at low mean TES latency times, of for example 11 ms, that TMS latency times originating from short detour loops involving axons arising from C2 and upper C3 regions would have gone undetected. Table 3 provides an overview of central MEP latency differences between ML–EL for TMS and TES for each case and mean of cases. The means of central ML-EL differences are in a range of 1.88–4.30 ms.

**TABLE 3:** Overview of MEP latency differences ML–EL of TMS and TES after separation of latency times originating from the indirect SAN conduction route.

ML – EL difference						
	
TES/TMS pairs	Left			Right		
	Mean ms	SD ms	*N*	Mean ms	SD ms	*N*
case 1	–	–	0	–	–	0
case 2	2.26	0.76	9	1.88	0.39	9
case 3	4.05	1.39	7	–	–	0
case 4	3.43	0.57	2	4.30	–	1
case 5	3.71	0.73	3^*^	3.97	0.61	4^*^

	**Both sides**					

	**Mean ms**	**SD ms**	**95% confidence interval ms**		**Range ms**	**N**
			
mean of cases	3.37	0.94	2.51 – 4.24		1.88 – 4.30	7

*Means are given case-wise and overall mean of all cases. The selection procedure is based on the application of case specific discriminating time borders being the sum of the mean trapezius latencies EL plus 4.5 ms to minimize false inclusion of indirect routes.*

*N is lower than in Table 1 due to replacement of the 18.5 ms general selection criterion by stricter case specific borders.

### Compound Axonal Motor Conduction Velocities

The low direct route fractions of TMS induced MEPs in Table 1 preclude reliable determination of MCV’s from TMS. In contrast, the near 100% fractions of latency times of TES provide the most reliable compound conduction velocities since these only pertain to the direct conduction route. The electrical compound velocities ECV in Table 4 result from the mean EL values of both sides (L&R) in Table 2.

**TABLE 4:** Overview of the mean and standard deviations (SD) of the overall (compound) motor conduction velocities for TES (ECV) across 12 cases comprising TES-MEP latency times EL of both sides with estimated fractions of the intradural length of the total axonal length of the direct route.

Trans-synaptic exclusion	ECV trapezius		ECV ECR		ECV TC	
	Mean m/s	SD m/s	Mean m/s	SD m/s	Mean m/s	SD m/s
none	64.01	7.21	66.17	4.68	73.67	4.38
2.5 ms synaptic delay (MN + NMJ)	77.46	9.34	76.70	5.44	79.27	4.72
4.0 ms synaptic delay (PN + MN + NMJ)	89.09	11.37	84.71	6.01	83.09	4.94

Estimated intradural fraction of the total axonal length of the direct route	**Dural exit**					
				
	**C2**	**C3**	**C4**			

	43%	55%	67%	58%		73%

*The conduction velocities are compound values of intra- and extradural (peripheral) conduction between vertex and trapezius muscles over an estimated total route length of ICL and ITL as depicted in Figure 1A. The intradural fractions between C2 and C4 vary between 43 and 67%. Total axonal conduction velocities are also given when excluding synaptic delays of 2.5 ms (neuromuscular junction and motoneuron) or 4.0 ms (additional proprioceptive neuron). The earlier published ECV values for the ECR and TC muscle groups of the same horses are mentioned for reference (Journée et al., 2020a).*

The ECVs of the trapezius are statistically equal to the ECVs of the ECR and TC of the same horses, which are also shown in Table 4. The estimated intradural fraction of the total length of the direct route at C3 level yields a close match with the intradural fraction of the ECR, as previously reported (Journée et al., 2020a).

### Dependence of Motor Evoked Potential Latencies and Amplitudes on Transcranial Electrical Stimulation and Transcranial Magnetic Stimulation Intensities

The dependence of transcranial MEP latencies and amplitudes of trapezius muscles of the transcranial stimulation intensity can be assessed statistically.

The dependence of the latency times on transcranial stimulation intensities is expressed by the slope coefficients of the regression lines for TES and TMS. All correlation coefficients were significant (<0.007). The slopes of the regression lines were for TES: –0.45 ms/10 V (left) and –0.35 ms/10 V (right) and for TMS: –0.88 ms/10% (left) and –0.43 ms/10% (right). In the supra-threshold ranges between 10 and 50 V and between 10 and 50%, the latency decreases were for TES –1.8 ms (left) and –1.4 ms (right) and for TMS –3.5 ms (left) and –1.8 ms (right). These values agree with the corresponding graphs in Figure 4.

### Comparison of Motor Evoked Potential Amplitudes for Transcranial Electrical Stimulation vs. Transcranial Magnetic Stimulation

**FIGURE 5:**
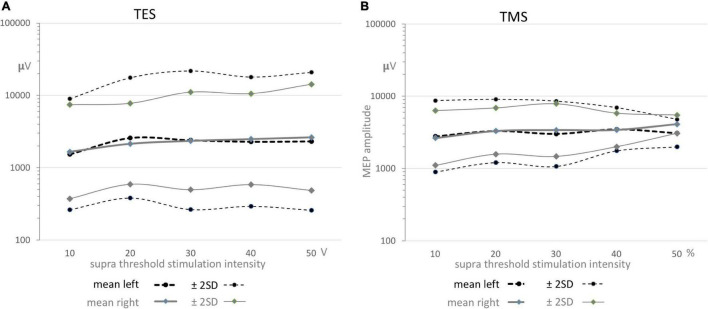
Functions of the geometric means ± 2SD confidence intervals as function of supra threshold transcranial stimulation intensities for TES **(A)** and TMS **(B)** for both sides. All graphs show a gradual small increase. The means in graphs A and B are nearly equal, while between graphs A and B the mean values are about 1 mV higher for TMS. The full coverage of the wide ± 2SD intervals in all graphs predict the absence of significance between means within TES or TMS and also between sides for both stimulation modalities as presented in Table 5.

Figure 5 shows for TES and TMS a relatively small increasing trend of MEP amplitudes with increasing supra threshold transcranial stimulation intensities. The means within graphs A and B are nearly equal to each other, while between graphs A and B the mean values for TMS are according to Table 5 about 1 mV higher than for TES.

**TABLE 5:** Survey of mean muscular MEP amplitudes for TMS and TES, indicated as MAMP and EAMP, respectively and their differences between TMS and TES for left (L) and right (R) sides and differences between sides (L-R).

	TMS MAMP		TMS-TES MAMP-EAMP		TES EAMP	
	Mean mV	sig	Mean mV	sig	Mean mV	sig
L	3.24		1.13	0.210	2.11	
R	3.22		1.00	0.765	2.22	
L-R	0.039	0.365			0.197	0.339

*The computations of means are performed in the logarithmic domain over all 5 TMS/TES cases and appear as geometric means after back-conversion to the linear domain. All differences are not significant for p < 0.05.*

The mean ± 2SD regions overlap each other almost completely. The about full coverage of the regions illustrate graphically the absence of significance in the differences between geometric mean amplitudes between sides and between TES and TMS which are summarized in Table 5. This means that MEP amplitudes are independent of transcranial stimulation modality and side.

### Central Conduction Velocities Measurements

Division of the enclosing segmental lengths between C2-3 and C5-6 of 32 cm and 30.6 cm by the multifidus TES-MEP latency differences of 1.78 ms and 2.09 ms (SEM: 0.13 ms and 0.26 ms) resulted in mean CCVs of 180 and 146 m/s (SEM 13 and 18 m/s), respectively.

## Discussion

Trapezius muscle MEPs offer the possibility to distinguish supraspinal lesions, including the C1 spinal segment, from spinal locations by functional assessment of the spinal cord and brain in horses. Furthermore, trapezius muscle MEPs enhance the differential diagnostic power to determine whether lesions are either focal or dispersed throughout the nervous system. The goal of the current prospective study was to provide normative data on MEP latency times and amplitudes of trapezius muscle MEPs elicited by TES as well as TMS in a group of healthy horses and to discover how the complex innervation *via* cervical roots and *via* the indirect SAN route becomes evident in practice and whether equine trapezius MEPs are useful for the proposed applications in practice.

The study revealed unexpected differences between TES and TMS creating new insights in the neurophysiology and neuroanatomy of the motor activation and innervation of trapezius muscles with implications for clinical neurophysiological practice.

### Normative Data of Motor Evoked Potentials

(A) Latency times:

The initial question whether direct conduction routes *via* cervical roots or indirect routes *via* the SAN to the trapezius muscle controls the MEP latency times could be answered. Table 1 shows for TES, the direct route fraction was represented for nearly 100% certainty in 120 measurements. This is the shortest and fastest route to the trapezius muscle and governs the latency time. Even when most of the motor activation is supplied along indirect SAN routes these fail in the competition with the direct straight routes because of their delayed arrival times.

The normative data for TES for both sides in 12 cases reveal TES MEP latency times ranging from 10.1 to 16.0 ms, with the exception of one 18.3 ms SAN outlier. According to Table 2 the mean±SD is 13.52±1.16 ms and the 95% confidence interval is 11.10–16.00 ms. The mean values of both sides are statistical equal to each other over a supramaximal TES intensity range of 10–50 V.

As opposed to TES, MLs from TMS clearly show mixed involvement of direct and indirect conduction routes (Figures 3B–E). This is reflected by a low direct route fraction that strongly varies between cases and by wider distribution functions of MLs, being about 2.5 times wider compared to TES (Table 1). These important differences between both stimulation modalities, TES vs. TMS, are also illustrated by the graphs of the means and confidence intervals in Figures 4A,B. The relative narrow EL distribution function between 10 and 16.5 ms is in marked contrast with the wide mean ± 2SD interval of TMS with MLs between 12 and 29 ms. This contrast is confirmed by the scattered normative data of the mean MLs and about 3 times larger SD values in Table 2. The high ML values remain within the transcranial time window below the range of latency times of extracranial elicited reflexes that are evoked by TMS.

The differences between TES and TMS obviously result from at least partly disjointed collections of recruitment of motoneurons. TES addresses always the direct route with recruitment in a restricted pool of motoneurons. Specifically TMS shows time varying selections of motor axon groups that belong at one time to direct and at other times to indirect routes.

It is concluded that TES is the most reliable and accurate stimulation method for clinical assessment of MEP latency times of trapezius muscles for which TMS should be considered obsolete because of the unpredictable involvement of direct or indirect conduction routes.

The trapezius muscle offers the possibility to differentiate between intracranial located lesions including the C1 spinal segment of the myelum at one hand and at the other hand the remaining spinal cord from C2 onward. Just like ECR and TC muscle MEPs, trapezius MEPs are easy to obtain. These superficial muscle groups are easy to locate and accessible for application of extramuscular electrodes. High cervical paraspinal muscle groups can also be used as alternative, however, they are more complex to access. These muscle groups are deeply localized and require an invasive placement of long intramuscular electrodes under ultrasonographic guidance by a skilled practitioner or radiologist. Attention should be paid to the fact that accurate EL measurements of TES rely on good signal quality with low background noise to prevent masking of sometimes low MEP amplitudes from a small fraction of cervical nerve axons. Because of guaranteed low impedances with a high success rate, subcutaneous electrodes are advised instead of adhesive surface electrodes (Journée et al., 2020b).

An alternative choice is the sternocleidomastoid (SCM) muscle. Like the trapezius muscle, the SCM is superficially located and easy accessible for extramuscular electrodes. The SCM is like the trapezius double innervated *via* cervical nerves and the SAN. However, the nerves represent the lower part of the medulla oblongata and cervical segments at the level of C1 and C2. In the rat, the columns of motoneuron somata are divided into three sections: a central column extending from the lower part of the medulla oblongata to and including C2, a ventrolateral column covering C2 and the upper half of C3 and a dorsomedial column covering C1 and C2 (Ullah et al., 2007). Except for the ventrolateral column, which supplies trapezius muscle groups further caudally, the other two columns already end below C2 level. Because of involvement of these clearly delineated high cervical locations it is expected that SCM MEP latencies will sharper distinguish between supraspinal and spinal located lesions while latency delays from indirect routes will be limited to maximal ∼3 ms for PCVs of around 90 m/s.

**FIGURE 6:**
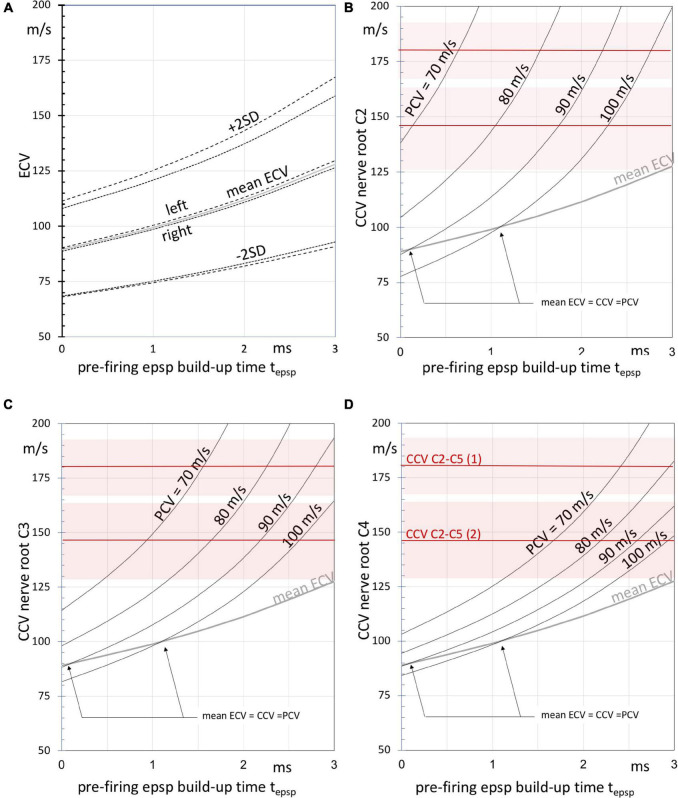
**(A)** Graphs of the left, right and estimated overall mean (dashed, dotted and gray lines, respectively) ± SD compound motor conduction velocities ECV after PN + MN + NMJ synaptic delay exclusion according to Table 4 as function of the build-up time t_epsp_, of epsp’s until firing after the start of TES. The ECV values result from the TES muscle MEP latency times. Panels **(B–D)** depict curves of the estimated (intradural) central motor conduction velocities (CCV) at three cervical nerve levels C2 (panel **B**), C3 (panel **C**), and C4 (panel **D**) as function of t_epsp_ using the estimated intradural fractions of the direct routes. The curves are given at peripheral motor conduction velocities (PCV) between 70 and 100 m/s. Note that higher CCVs are associated with lower PCVs and vice versa to outbalance the ECV values. The gray curves represent the mean ECV. The arrows indicate intersecting points at which mean ECV values are equal to peripheral and central motor conduction velocities. The horizontal red lines represent mean CCVs as derived from the multifidus MEP latencies enclosing the segmental region between cervical nerves C2 and C5 in two horses, numbered (1) and (2). Shaded areas represent the SEM.

The TES intensity dependent reductions of MEP latencies of –1.4 to –1.8 ms over supramaximal intensities up to 50 V and –1.7 to –3.5 ms over 10–50% for TMS are comparable with those reported for the ECR and TC MEPs in the same horses (Journée et al., 2020a).

(B) Compound and central axonal conduction velocities:

The estimated mean (± SD) overall compound muscle conduction velocity (ECV) for TES between vertex and trapezius on both sides is 64.0 (± 7.2)m/s and includes both central and peripheral axons of the direct route. The net axonal conduction time can be deduced by correcting for synaptic delays originating from the neuromuscular junction, peripheral motoneuron and proprioceptive neurons as described previously (Journée et al., 2020a). After synaptic delay correction the mean (± SD) ECV is 89.1 (± 11.3)m/s with a 95% interval of 67–111 m/s. Table 4 shows an overlap with previously reported ECVs of extensor carpi radialis (ECR) and tibialis cranialis (TC) muscles of the same horses. The build-up time t_epsp_ is not included in the corrections for synaptic delays. This build-up delay depends on the state of facilitation which for TES and TMS is maximal 3 ms when starting-off from a fully relaxed muscle (Rossini et al., 2015). The correction for the epsp build-up time is applied in addition to the synaptic delay correction and is shown in Figure 6A as mean ± 2SD of the corrected ECV as function of t_epsp_.

It is noted that ECVs are compound values. These are weighted averages of central and peripheral conduction velocities. When the central (intradural) fraction of the direct route is known, it is possible to derive the central conduction velocity CCV from the ECV. The relationship between these parameters is based on the assumption: EL = CT + PT, where the latency time EL is the sum of the conduction time CT over the central route length CLd and the conduction time PT over the peripheral route length PLd. This pertains only to the direct route as depicted in Figure 1B. The CCV is calculated after substitution of EL = (CLd + PLd)/ ECV, CT = CLd/CCV and PT = PLd/PCV. CLd is linear related to the intradural fractions of 43–67% of the C2, C3 and C4 routes in Table 4. The intradural fraction of 55% of C3 matches most with the 58% of the conduction route reported for the ECR (Journée et al., 2020a). The high fraction of 73% of the TC is most closely approached by the 67% of C4. PCVs in horses are published for median and radialis nerves. Proximal PCV values from stimulation at the plexus brachialis are 96.4–100 and 86.8–90.2 m/s, respectively, and at distal stimulation 80.7–88 and 71.1–79.5 m/s (n. medianus and n. radialis, respectively) (Henry et al., 1979). Parameter values of PCV = 70–100 m/s are selected for computations of the CCV functions in Figures 6B–D. The functions are useful for qualitative comparisons to describe effects caused by changes of parameters. At given ECV compound velocities, higher CCVs outbalance lower PCVs and vice versa with transition points at ECV = PCV = CCV. For PCV = 90 m/s and t_epsp_ = 2 ms, the graphs predict CCVs between 125 (Figure 6D) and 165 m/s (Figure 6B) for direct routes along C2 to C4. The higher CCVs of C2 are explained by the longer peripheral axonal length PLd of C2 when compared to C4. This means longer exposure times to low PCVs with longer delays. These need extra compensation by high CCVs that have to catch-up along the shorter central axon route length CLd of C2. CCVs incline indeed most rapidly at the C2 route as depicted in Figure 6B, representing the shortest intradural length fraction. The epsp build-up time corrections represent a hyperbolic function, which becomes inaccurate in proximity of an asymptote. This may result in unrealistic high ECV values. This is the case for C2 nerve routes in Figure 6B. The CCV bias is highest for PCV = 70 ms (asymptote: t_epsp_ = 2.2 ms) and lower for PCV = 80 m/s (asymptote: t_epsp_ = 3.0 ms). The accuracy of estimated CCVs improves further away from the asymptotes.

The most reliable measurements of the intradural motor conduction times and ECV from muscle MEPs are expected at highest intradural length fractions, which entails for the trapezius muscle the C4 nerve root level. From the curves in Figures 6B–D it is likely that intradural conduction velocities of axons are well above 100 m/s and may reach 180 m/s.

The mean CCVs of the two horses of 180 (SEM 13)m/s and 146 (SEM 18)m/s comply with this range. The multifidus muscle, which is an epaxial muscle, connects 2 subsequent spinal vertebral bodies and is monosegmentally innervated by branches of the dorsal spinal nerve belonging to the metamere of its cranial insertion point (García Liñeiro et al., 2017). This muscle is therefore considered to offer optimal segmental selectivity among other paraspinal muscles. According to the Hursh factor, an axonal conduction velocity of 180 m/s would predict presence of axon diameters of 30μm in motor tracts of the myelum (Hursh, 1939). Our data comply with literature. Similar CCVs can be deduced from the data reported for segmental TMS-MEP latency times of intertransversarii MEPs in the paper of Rijckaert et al. (2018). These are paraspinal muscle groups which are examined by placing EMG needle electrodes laterally from the cervical bodies. These muscle groups are expected to be innervated from the cervical nerve cranial from the corpus. It is unsure if the innervation may be considered as monosegmental. In the latter study significant differences between mean latency times of male horses vs. mares mare were reported. The extracted MEP latency differences between corpora C3 and C6 yielded for mares 2.0 ms and for male horses 2.8 ms, respectively. When using the mean lengths across the 3 segments from our data and applying a coefficient of variation of 10%, the estimated mean velocities and 95% confidence intervals is for mares 156 (125–187)m/s and for male horses 115 (93–137)m/s. The CCVs of the 2 mares in our study agree with the range for mares. It has to be mentioned that the CCVs of male horses will ratio-wise be higher when their larger corpus lengths are taken into account.

(C) Motor evoked potential amplitudes:

Figures 5A,B show for TES and TMS for both sides an equal evolvement of mean amplitudes as function of supramaximal transcranial intensities with initially a minor increase reaching a plateau after the first intensity step. The full overlap of the wide ± 2SD intervals in all graphs predict the absence of significant correlation between TES or TMS and also between sides for both stimulation modalities. This is statistically supported in Table 5. It is concluded that there are no statistical differences between sides and modalities with respect to MEP amplitudes. As expected, the direct route *via* cervical root nerves or the indirect route of the SAN play no role in the mean amplitude values that have been recorded within the transcranial time windows.

However, as deduced from the logarithmic plots, normative data show differences between the widths of the 95% confidence intervals from TES and TMS. The differences in the logarithmic domain were translated to ratios in the linear domain. The normative values for widest parts for TES are between 0.26 and 22 mV (ratio 1:83) for left and between 0.5 and 15 mV (ratio 1:28) for right, while for TMS the widest parts are enclosed between 0.9 and 9.1 mV (ratio 1:10) for left and 1.1–7.9 mV (ratio 1:7) for right. The TES-TMS differences between the 95% interval ranges are clearly reflected in these ratios. We have no explanation for the differences. It is noted that the TMS data result from 5 cases, which should be considered as a small number.

### Differences Between Transcranial Electrical Stimulation vs. Transcranial Magnetic Stimulation-Motor Evoked Potentials

The significant finding of the current study is the presence of a large variability of trapezius MEP latency times for TMS as visible in the wide dispersed histograms in Figure 2 where the variability is not only present in repeated measurements within one muscle group, but also between muscle groups and between cases.

Important to notice is the absence of recorded trapezius muscle responses (M-waves) in the current study from direct activation of peripheral nerves by extracranial conducted stimulation pulses. These would be recognized as highly reproducible relative narrow muscle responses and distinguishable short latency times. The longest latency times of M-waves are expected at direct nerve stimulation most distant from the trapezius muscle in a region just caudal from the head. When using the measured inion-trapezius electrodes distances together with PCV = 90 m/s, expected longest M-wave latencies are 7.2 ± 1.7 ms (mean ± SD). This is below the shortest trapezius TES-MEP latency time of 10 ms.

(A) Identified locations of active motoneurons in the cervical myelum:

As mentioned previously, differences in latency times along the indirect route permit to estimate the location of active motoneurons from which these result. Figure 2 is an impressive illustration of how within a single case (case 1) trapezius latencies can differ between TES and TMS, and for TMS between sides. Of the illustrated case, all TMS latency times ML represent the indirect route. The locations of the active motoneurons from the apex in the spinal cord d_apex–MN_ can be estimated from latency differences between indirect and direct routes according to equation 1. Implementation of a TMS-TES correction of ML-EL = 3.37 ms (Table 3), yields: d_apex–MN_ = PCV. (ML-EL-3.37 ms)/2. Using the mean latency pairs (ML, EL) of case 1 for left: (20.34 ms, 11.458 ms) and for right: (25.02 ms, 11.60 ms) with PCV = 90 m/s, d_apex–MN_ is 24.67 and 45.09 cm, respectively. The TMS activated motoneurons reside for the left and right sides on different locations at 2.5 and 4.5 segmental levels caudal from the apex at a segmental length l_seg_ of 10 cm, estimated as 1/7th of the 70 cm neck length of this horse.

ML values allow to estimate the caudal extend of active MNs. The largest measured ML of the 5 TES/TMS cases is 27.23 ms. Using the previous approach with PCV = 90 m/s and l_seg_ = 10 cm this ML indicates that the by TMS activated motoneurons overlay 4.93 ± 0.68 segmental levels from the SAN apex.

The estimated range of motoneuron locations from the TMS MEP latencies stretching out over 5 segments complies with the neuro anatomic range of many animals and humans between the lower part of C1 or caudal three-quarters of C2 to the rostral quarter of C6 (Routal and Pal, 2000; Ullah et al., 2007). In sheep, the trapezius motoneurons are located in a single ventrolateral column from C2 to even C6 (Clavenzani et al., 1994). This was assessed using retrograde tracing techniques. Boehm and Kondrashov (2016) reported for 15 SANs of 8 human cadavers the following supply of rootlets: 5 from C1-C4, 8 from C1-C5 and of 2 from C1-C6.

To our knowledge, the approach used for neurophysiological tracing of the segmental location of active trapezius motoneurons by means of TES or TMS applied in the current study is new and adds an important functional insight into the neuro-anatomy of the motor function and innervation of the trapezius muscle in horses which may be of clinical diagnostic importance.

(B) Differences in motoneuron recruitment and functionality of nerve elements in the cervical plexus:

As depicted in Table 1, cervical nerve roots are partly activated when TMS is applied. The about 100% success rate of TES for MEP latency times indicates that at least one of these cervical nerves is always functional. Transcranial stimulation causes a subtotal recruitment of motoneurons, mostly below 10%. This leaves the opportunity for activation of disunited motoneuron pools and facilitates occurrence of stochastic variations in time and segmental location involvement as illustrated by the MEP latency times of TMS.

Supramaximal direct nerve stimulation activates all myelinated axons in a nerve and is used to check the contribution of each cervical root and SAN to the innervation of the trapezius. This approach is applied intraoperatively for the purpose of surgical decision making as to which nerve can be dissected. All studies recognize an important role of the SAN and show varying anatomical and neurophysiological contributions of the cervical nerves of C2, C3, and C4, of which not necessarily all are functional. Gavid et al. (Gavid et al., 2016) showed in 12 human cadaver dissections presence of about 78% communicating branches between the SAN and C2, 48% between SAN and C3 and 52% between the SAN and C4 and analyzed retrospective data of intraoperative electroneurography studies from 13 patients undergoing 25 modified neck dissections. Selective supramaximal stimulation elicited trapezius MEPs in 7% of C2 stimulations, 20% of C3 and also 20% in C4, whereas all SAN stimulations resulted in contractions of all 3 parts of the human trapezius muscles. Similarly, Pu et al. (2008) performed selective stimulation in 34 patients undergoing a radial neck dissection of the SAN and C2-C4 branches and reported a success percentage of successful trapezius responses of 100% for the SAN, 44% for C2, 62% for C3 and 59% for C4. Likewise, Kierner et al. (2002) reported in 17 modified radical neck dissections performed on 14 patients recordable MEPs in all SAN stimulations, but did not report on results of cervical root stimulation. Zhao et al. (2006) performed non-selective supramaximal stimulation of the cervical plexus in 18 rats and looked into the effects of transections of the SAN (group A), the C2-C5 trunks (group B) and transection of both SAN and the C2-C5 trunks (group C) on trapezius contractions. They concluded that the SAN is the most important contributor, while motor innervation provided by the cervical plexus was considered as not very significant though not absent. Krause et al. reported considerable inter- as well as intra-individual differences in 47 post-mortem dissected human bodies in which the SAN was mostly, but not always involved in the innervation of the trapezius (Krause et al., 1991).

The aforementioned studies illustrate the presence of substantial variations in the double innervation of the trapezius muscle groups, which are seen between species, as well as within species. The bottom line is that the major supply runs *via* the indirect route, which in most cases, but not always, reclaim responses in 100% of SAN stimulations. In contrast, these percentages are much lower (between 0 and 59%) for individual cervical roots located between C2-C4. Much higher percentages are expected when motor responses can be obtained when more roots are stimulated. These functional data are to our knowledge not available in horses.

The high success rate of about 100% reported in the current study of short TES-MEP-latencies that are expected from direct conduction *via* the cervical roots reflect that in horses, at least one of the C2-C4 roots is functional. The lower success percentage of TMS indicates that a part of the TES pool of recruited motoneurons that supply cervical nerves to the trapezius muscle is excluded. TMS predominantly activates cortical axons (Amassian et al., 1989; Edgley et al., 1990; Burke et al., 1993). The lower success rate of TMS for activation of the direct route can possibly be ascribed to the involvement of the processing role of pyramidal neurons. In the pyramidal system, motor potentials become distributed across axons of different diameters in concordance with the Hennemans principle (Henneman et al., 1965). This makes it unlikely that all large fast pyramidal axons are addressed. This would exclude the full recruitment of pyramidal axons, which can be achieved by application of supramaximal TES. The full recruitment that is achieved by TES is obviously a critical condition for activation of the direct cervical route.

### Study Limitations

- The large variability in direct and indirect conduction route involvement seen for TMS challenges the statistical power. A larger study group would have been more suitable to further pinpoint the direct vs. indirect route fraction involvement when applying TMS.
- The accuracy of the model for prediction of the intradural axonal conduction velocities depends on the unknown state of muscular facilitation, which controls the pre-motoneuron firing epsp build-up time.
- The variability seen for of active trapezius motoneuron involvement between C2 and C5 limits the spatial sharpness to localize lesions in high cervical regions.

### Study Highlights

In horses:

- TMS and TES cannot be considered as equivalent techniques for clinical diagnostic use of latency times of trapezius MEPs
- Trapezius MEP latency times from TES represent the fastest conduction route along cervical nerves with a high success rate, provide solid normative data and confirm in all horses functional presence of cervical roots.
- In contrast to TES, TMS delivers a large variability of latency times due to unpredictable contributions of direct and indirect nerve routes to trapezius muscles, leaving TES as the only reliable modality for clinical diagnostic use.
- The large variation width of TMS trapezius MEP latencies of up to ∼15 ms from the SAN route indicate locations of active motoneurons between C2 and C5 and possibly the upper quarter of C6 and enclose at least 5 cervical segments. The locations may differ between sides.
- Long TMS-trapezius MEP latency times don’t interfere with MEPs from extracranially elicited reflexes.
- Predicted from trapezius latencies and verified by multifidus MEP latencies, intradural motor conduction velocities may in some horses reach 180 m/s forecasting maximum axon diameters of 30μm.

## Conclusion

This is the first study to report on normative data for both TES and TMS induced MEPs of trapezius muscles. In contrast to MEPs from limb muscles in the same horses where TMS and TES are interchangeable modalities, the complex innervation of the trapezius muscle leaves TES as only reliable alternative for clinical diagnostic use. This statement is based on the observation that in TES axons of the fastest straight conduction route along cervical nerves always participate, which is in contrast to TMS where delayed arrival times from SAN routes often become evident. The applied model shows that SAN route lengths and the wide dispersed latency times are governed by locations of involved motoneurons over C2-C5 segments. Long TMS trapezius MEP latencies don’t interfere with extracranial elicited reflexes. TES intensity dependent reductions of trapezius MEP latencies are similar to those reported for the ECR and TC while MEP amplitudes between sides and between TES and TMS are not different. Intradural motor conduction velocities may in some horses approximate 180 m/s forecasting maximum axonal diameters up to 30μm.

## Data Availability Statement

The raw data supporting the conclusions of this article will be made available by the authors, upon reasonable request.

## Ethics Statement

The animal study was reviewed and approved by the Animal Ethics Committee of the University of Groningen, the Netherlands the study was registered as DEC6440A and DEC6440B. Written informed consent for participation was not obtained from the owners because the owners agreed verbally for their horse to participate in the study.

## Author Contributions

SJ and HJ contributed equally to this article in study design, application for ethical approval, data acquisition and analysis, and interpretation and writing. HB, SR, CD, and WB critically revised important for the intellectual content based on their professional background. CB contributed to the provision of experimental facilities and giving practical advices. CD involved in concluding stage regarding interpretation, and writing and revision. All authors contributed to the article and approved the submitted version.

## Conflict of Interest

The authors declare that the research was conducted in the absence of any commercial or financial relationships that could be construed as a potential conflict of interest.

## Publisher’s Note

All claims expressed in this article are solely those of the authors and do not necessarily represent those of their affiliated organizations, or those of the publisher, the editors and the reviewers. Any product that may be evaluated in this article, or claim that may be made by its manufacturer, is not guaranteed or endorsed by the publisher.

## Abbreviations

ACL_e_: accuracy of TES-MEP latency
ACL_m_: accuracy of TMS-MEP latency
d_apex–MN_: distance apex–active motoneuron
EAMP: TES-MEP amplitude
ECR: m. extensor carpi radialis
ECV: TES compound conduction velocity
EDM: equine degenerative myeloencephalopathy
EL: TES-MEP, latency
epsp: excitatory postsynaptic potential
ET: TES motor threshold
MAMP: TMS-MEP amplitude
MCV: TMS compound conduction velocity
MEP: motor evoked potentials
ML: TMS-MEP latency
MT: TMS motor threshold
PN: proprioceptive neuron
SAN: spinal accessory nerve
TC: m. tibialis anterior
Td: MEP latency time direct route
t_epsp_: build-up time of epsp’s from onset to firing threshold of motoneurons
TES: transcranial electrical stimulation
TMS: transcranial magnetic stimulation
Ti: MEP latency time indirect route.
